# Remarks on the Disease of Which Dr. Rush Died

**Published:** 1817-06

**Authors:** 


### *For the London Medical and Physical Journal.*

### *Remarks on the Disease of which Dr. Rush died;* by H. E.

YOUR Number for this month contains a letter from Dr. Mease, of Philadelphia, to the late Dr. Lettsom, concerning the disease of which Dr. Rush died. I have been a good deal struck with several passages in that letter, on which I shall presume, with your leave, to make a few remarks,—with no intention whatever of cavilling or quibbling, but purely for the sake of obtaining information.

There is no man of liberal sentiments but must join Dr. Mease and you in the merited censure which you pass on those contemptible spirits, who, for the purpose of gratifying their malignant passions, are ever ready to view the unfavourable side, and to vilify professional reputation. Fortunately, in this instance, their triumph has been but short-lived; for, whatever reflections may arise out of the history of the case, it is sufficiently clear that the medical sage of Philadelphia did not fall a sacrifice to the depletory system of treatment.

In proof of this fact, Dr. Mease informs us that Dr. Rush "was not afflicted with *typhus* or *spotted fever*, but with a true pleurisy, and the blood, so far from being *freely* taken, amounted only to *ten* ounces in quantity; more was not taken away (except locally), although the pain in his side, after having been relieved by the operation, returned with severity, and the disease ended as inflammatory affections of the lungs often do in such habits as that of Dr. Rush.

This, however, is not the principal point to which I direct your attention. We are told by Dr. Mease, in his explanation of the treatment, that the taking away of the ten ounces of blood *certainly weakened him*. Now, sir, it is this piece of information which perplexes me, and tends to confound all my notions respecting inflammatory diseases and blood-letting. I do not profess to know precisely the many local circumstances which may modify climate and other causes that produce disease in the city and neighbourhood of Philadelphia; but Dr. Mease has furnished us with one point of parallelism between that situation and the British islands, by stating that the climate is "inconstant." So far, we may presume, there is a certain similarity, and venture to reason accordingly. Can we suppose, then, that a loss of blood to the amount of *ten* ounces, in a *true pleurisy*, of not more than twelve hours' continuance, and in a patient who is described as having been "as well as usual through the day" before, before, could have *certainly weakened?* If this be the case, I must acknowledge, with surprise and sorrow, that all my ideas on the subject are completely unhinged. If, at the commencement of one of those diseases the most decidedly inflammatory and fatal, the balance be so delicately adjusted, that, under the circumstances stated in this history, the abstraction of ten ounces of blood does *certainly* weaken, who shall any more hazard to prescribe or to employ the lancet?

But such an opinion is so greatly at variance with all the more commonly received views of the subject, at least on this side of the water, that one of two conclusions, I humbly submit, must inevitably follow from the account: Either that the disease of which Dr. Rush died was not a true pleurisy, in which case, the loss of ten ounces of blood might possibly, though, I think, not at all probably, do harm; or, allowing it to have been a true pleurisy, which there appears every reason to believe that it was, the ten ounces of blood could not by possibility be hurtful. Really, thus much would seem to be absolutely necessary, if it be wished to preserve confidence or consistency in medical theory and practice. For, making every allowance for difference of age, constitution, and situation, if we find, in one page of your Journal, that, in the variable climate of Philadelphia, the loss of ten ounces of blood, in a true pleurisy, produced weakness; and a few pages farther of the same Number of your Journal, that " early bleeding, not regulated by *quantity* but by *effect*, was the remedy, and, in some cases, that 200 oz. were abstracted in two or three days, besides *other* evacuations and cold affusions," and that too in fever, and in a tropical climate, where and how shall we discover a criterion by which to regulate our practical measures? In fact, there can be no longer any guide, and " chaos is come again."

I am sorry, therefore, to be under the necessity of differing entirely with so respectable an authority as Dr. Mease, on this particular point of the case, and of concluding, that, whatever had occasioned the debility in his venerable friend, it could not have been the loss of so small a quantity of blood. Indeed, an impression widely different has been left upon my mind by the perusal of this interesting case. I am irresistibly led to imagine, that it was not the loss of these poor ten ounces of blood that produced any such effect, but the not having *early* lost ten, or twenty, or thirty, ounces *more*, or at least such a quantity as would have made some salutary and decisive impression on the system; thus affording the disease (supposing it still to be true pleurisy) full leisure to establish establish itself, and, by that means, *certainly* to *weaken* the patient. I make this observation with the more freedom that this part of the treatment was the suggestion of Dr. Rush himself, consequently no living practitioner can well be implicated in the omission. What particular features the symptoms assumed so as to deter from pursuing the plan of evacuation farther, it would be presumptuous to conjecture, as we are provided with no grounds upon which to proceed. Besides, those gentlemen who were consulted were fully competent to form an opinion, and, in fairness and candour, we must, of course, admit that their judgment was a correct one. But, if any farther evidence were requisite that the blood-letting did not *certainly weaken*, it is to be found in the statement of Dr. Mease, and in the more detailed account of Mrs. Rush, on which that statement is founded. The former acquaints us with the fact of the pain in Dr. Rush's side "having been relieved by the operation" of blood-letting; and Mrs. R. says, that the blood was taken from his arm " with evident relief," that " he remained the rest of the day (Thursday) and on Friday with but little apparent disease,"—and that " on the pain of his side, which had on Saturday returned acutely, still continuing, and his breathing becoming more difficult, he was so relieved by the loss of three ounces of blood by cupping, to which Dr. Physick had consented, that he fell into a refreshing sleep, passed a comfortable night, and on Sunday morning seemed free from disease."

Again, Dr. Mease, in explaining the practice, admits that, "though the blood-letting certainly weakened, yet had it not been ordered, it is highly probable that the pain of his side, and the great difficulty of breathing, which showed pulmonary congestion, would have exhausted him even before the period at which the disease finished its course." It in all probability arises from the dulness of my apprehension, but I cannot, for the life of me, perceive the satisfactory clearness of this reasoning. How could the blood-letting be said to weaken, when, by the very account, it so immediately and completely relieved the pain in the side, that is, the inflammation, which was the cause of the pulmonary congestion and the difficulty of breathing? How could that be said actually to debilitate, which protracted the patient's life? However debilitating the abstraction of blood, that fluid upon which the perfect performance of all the functions so essentially depends, may be, considered in relation to the body in the healthy state, yet, in relation to the body in a state of disease, it may prove directly strengthening. If it diminishes the force of that power, viz. the disease, which is is already rapidly weakening the vital energy, and which, if allowed to proceed uninterrupted in its operation, must speedily cause death, the effect is any thing but debility.

I have taken the liberty of troubling you thus minutely, as the celebrity of the parties concerned, the interest which the medical public has doubtless felt in the circumstances detailed, and the publicity which has been given to them, seem to leave the subject open to observation. But, above all, it is a matter of the highest importance that correct and well-defined notions should prevail on points, which so seriously concern the conduct and reputation of practitioners, as well as the lives of their patients.

*May* 9, 1817.

---

**We have given insertion to this very candid inquiry for many reasons, but, most of all, because we wish it to be understood by our younger readers, that, even admitting the patient felt weaker after bleeding, this is by no means a sufficient objection to the repetition of that practice, if pain and other symptoms of inflammation returned. Nor is it any proof that the weakness arose from loss of blood. Inflammation is itself a violent action, and leaves the subject weaker; but it also, when carried beyond a certain extent, destroys the function of an organ, or kills the patient. It must, therefore, be subdued at all events.—Edit.**

**N.B.—This remark is not written by the gentleman personally addressed in this communication.**

